# Cell fate determinant Llgl1 is required for propagation of acute myeloid leukemia

**DOI:** 10.1038/s41375-023-02005-9

**Published:** 2023-08-16

**Authors:** Theresa Eifert, Chen-Jen Hsu, Alicia L. Becker, Sarah Graessle, Arik Horne, Franziska Bemmann, Qirui Zhang, Michael Heuser, Valeri Vasioukhin, Sebastian Scholl, Andreas Hochhaus, Florian Siegerist, Nicole Endlich, Lars Bullinger, Steven W. Lane, Simon Haas, Tina M. Schnoeder, Florian H. Heidel

**Affiliations:** 1 Innere Medizin C, Universitätsmedizin Greifswald, Greifswald, Germany; 2 Charité-Universitätsmedizin, 10117 Berlin, Germany; 3 Berlin Institute of Health (BIH) at Charité - Universitätsmedizin Berlin, 10117 Berlin, Germany; 4 Berlin Institute for Medical Systems Biology, Max Delbrück Center for Molecular Medicine in the Helmholtz Association, 10115 Berlin, Germany; 5 Humboldt-Universität zu Berlin, Faculty of Life Sciences, Unter den Linden 6, 10099 Berlin, Germany; 6 Department of Translational Oncology, National Center for Tumor Diseases (NCT) Heidelberg, German Cancer Research Center (DKFZ), Heidelberg, Germany; 7 Hematology, Oncology, Hemostaseology and Stem Cell Transplantation, Hannover Medical School (MHH), Hannover, Germany; 8 Division of Human Biology, Fred Hutchinson Cancer Research Center, Seattle, WA USA; 9 Innere Medizin II, Universitätsklinikum Jena, Jena, Germany; 10 Institute of Anatomy and Cell Biology, University Medicine Greifswald, Greifswald, Germany; 11 QIMR Berghofer Medical Research Institute, Brisbane, QLD Australia; 12 The University of Queensland, Brisbane, QLD Australia; 13 Royal Brisbane and Women’s Hospital, Herston, QLD Australia; 14 Division of Stem Cells and Cancer, Deutsches Krebsforschungszentrum (DKFZ) and DKFZ – ZMBH Alliance, Heidelberg, Germany; 15 Heidelberg Institute for Stem Cell Technology and Experimental Medicine (HI-STEM gGmbH), Heidelberg, Germany; 16 German Cancer Consortium (DKTK), 69120 Heidelberg, Germany; 17 Leibniz Institute on Aging, Fritz-Lipmann-Institute, Jena, Germany

**Keywords:** Translational research, Acute myeloid leukaemia

## Abstract

Scribble complex proteins can influence cell fate decisions and self-renewal capacity of hematopoietic cells. While specific cellular functions of Scribble complex members are conserved in mammalian hematopoiesis, they appear to be highly context dependent. Using CRISPR/Cas9-based genetic screening, we have identified Scribble complex-related liabilities in AML including *LLGL1*. Despite its reported suppressive function in HSC self-renewal, inactivation of *LLGL1* in AML confirms its relevant role for proliferative capacity and development of AML. Its function was conserved in human and murine models of AML and across various genetic backgrounds. Inactivation of *LLGL1* results in loss of stemness-associated gene-expression including *HoxA*-genes and induces a GMP-like phenotype in the leukemia stem cell compartment. Re-expression of *HoxA9* facilitates functional and phenotypic rescue. Collectively, these data establish *LLGL1* as a specific dependency and putative target in AML and emphasizes its cell-type specific functions.

## Introduction

The Scribble protein complex has been discovered and characterized in drosophila [1]. Complex members serve as scaffold proteins and regulate cell polarity, motility and growth mainly through protein–protein interactions. In drosophila models, genetic inactivation of either Scribble complex member (*Lgl*, *Scrib*, *Dlg*) led to neoplastic tissue overgrowth supporting their role as one of the first described tumor suppressors [2]. Recent data suggest involvement of Scribble polarity complex proteins in regulation of HSC biology [3, 4], immune cell function [5] and potential implications in development of hematopoietic cancers [4, 6, 7].

Genetic inactivation of *Llgl1* was associated with an increase of long-term (LT-) HSC numbers and these cells showed competitive advantage when transplanted serially into recipient mice [4]. *Llgl1* deletion by itself did not cause leukemia, however, its expression was correlated with decreased survival in AML. Recently, mutations of *Llgl2*, a close human homolog of *Llgl1*, have been described as an early genetic event in progression from severe congenital neutropenia to AML [8]. In contrast, tumor suppressor function of *Llgl1* was not conserved in murine models of lymphoid (B- and T-cell) leukemia [9]. Deletion of *Scrib* resulted in impairment of long-term HSC function [7] and also affected proliferative capacity of AML. So far, data on the function of *Dlg* and its human homologs in hematopoiesis and leukemic transformation is lacking.

While specific cellular functions of Scribble complex members appear to be conserved in mammalian hematopoiesis, they seem to be highly context dependent. Differentiation stage, lineage commitment, underlying genetic background, the mechanism of genetic inactivation and the underlying cellular state may contribute to a highly variable phenotype. In this study, we screen for the requirement of Scribble complex members in AML. Unexpectedly, we identify *Llgl1* as a functional vulnerability. We address the issue of context dependent effects of *Llgl1* inactivation in models of acute myeloid leukemia and provide evidence that *Llgl1* is functionally required for maintenance of an undifferentiated state and proliferative capacity of AML.

## Material and methods

### Cell lines and culture conditions

Cell lines were purchased from DSMZ (Braunschweig, Germany). Cells were cultured according to standard protocols and tested negative for mycoplasma. For proliferation assays, the number of cells was counted following trypan blue exclusion. Apoptosis was measured by flow cytometry using Annexin V/SYTOX^®^ Blue staining at day 6 post-infection. Cell cycle analysis was performed at day 14 post-infection using the Click-iT EdU Kit (Life Technologies, Darmstadt, Germany) following the manufacturer’s instruction.

### Methylcellulose colony-forming assays

Primary human cells isolated from peripheral blood of AML patients were seeded in methylcellulose according to standard protocols and as published previously [10].

### Animal models

All experiments were conducted after approval by the Landesverwaltungsamt Sachsen-Anhalt (42502-2-1052 UniMD) and the TLV Thüringen (02-035/16). FLT3^ITD/ITD^ mice were a gift of Prof Benjamin L. Ebert (Harvard Medical School, Boston, MA, USA) [11]. MLL-AF9 knock-in mice were a gift from Prof Terrence Rabbitts (Institute of Cancer Research, London, UK) [12]. Mx1-Cre (Strain 03556) mice were obtained from Jackson Laboratories. C57BL/6J mice (6–8 weeks old) were purchased from Janvier Labs (Le Genest-Saint-Isle, France) and housed in a pathogen-free animal facility. Mice harboring a ‘floxed’ (flanked with loxP sites) allele of *Llgl1* have been generated as previously described [13]. Exon 2 (the exon downstream from the exon with the first ATG codon) was flanked by LoxP sequences, and the β-geo selectable marker was removed by transient expression of Cre-recombinase in the ES-cells. These mice have been backcrossed more than 8 generations into a C57BL/6J background. For transplantation, mice were irradiated as indicated and transplanted via tail intravenous (IV) injection with 1 × 10^5^ to 10^6^ bone marrow as indicated. Mice were sacrificed and analyzed at a defined time-point or when signs of disease became evident. Disease burden was assessed by complete blood counts, flow cytometry of peripheral blood (PB), bone marrow (BM) and spleen cells or histopathological stainings. To activate Mx1-Cre in vivo, poly(I):poly(C) (Cytiva, Marlborough, MA) was injected two times every second day intraperitoneally. Injections were halted if mice showed signs of illness prior to completion of treatment. Spontaneous Mx1-Cre activation was noted as previously described consistent with spontaneous activation of Mx1-Cre in an inflammatory milieu [14]. NOD-Prkdcscid-IL2rgTm1/Rj (NXG) mice were obtained from Janvier Labs (Le Genest-Saint-Isle, France) and NOD.Cg-*Prkdcscid Il2rgtm1Wjl* Tg(CMV-IL3, CSF2, KITLG)1 Eav/MloySzJ (NSGS) mice were obtained from The Jackson Laboratory (Bar Harbor, USA). AML cell lines (as indicated) or primary human xenografts were genetically modified by RNAi and subsequently injected at equal distribution into recipient mice. Therefore, no randomization was necessary. Due to the analysis in paired samples (cells transduced with either shRNA or non-targeting control), no blinding was necessary. Sample size and experimental schedule were calculated assuming a relevant difference in means of survival. We used a one-sided t-test at a = 0.05 and a power of >80% with an expected difference in means of 1.75 SD (standard deviations) based on previous experience with xenotransplantation. Equal numbers of 8–12-week-old male and female mice were used for experiments in all groups.

### Blood analysis and bone marrow cytospins

Blood was collected into EDTA-coated tubes and investigated using a BC-5000Vet (Mindray, China). To analyze cell morphology, 1 × 10^5^ bone marrow cells were centrifuged onto glass slides. Peripheral blood smears and bone marrow cytospins were stained with Wright-Giemsa (BioScientific).

### Histological imaging of mouse organs

Spleen, liver and lung were fixed and embedded according to standard protocols. Slides were automatically processed for hematoxylin and eosin staining (Leica AutoStainer XL, Leica Biosystems, Wetzlar, Germany). Images were acquired at 10× magnification on an AxioImager A.2 (Carl Zeiss Microscopy, Jena, Germany). Images were processed and analyzed using the ZEN software (blue edition, version 2.3, Carl Zeiss Microscopy GmbH, Jena, Germany).

### Flow cytometry

For immunophenotype analysis, peripheral blood cells, bone marrow or spleen cells were resuspended in PBS/1% FBS after erythrocyte lysis (PharmLyse^TM^, BD Pharmingen, San Diego, CA). Unless otherwise stated, the following antibodies were used: Sorting and analysis of LSK cells (Lin-Sca-1+cKit+) were performed as previously described [10, 15]. Biotinylated antibodies against Gr-1 (RB6-8C5), B220 (RA3-6B2), CD19 (6D5), CD3 (145-2C11), CD4 (GK1.5), CD8 (53-6.7), TER119 and IL7Ra (A7R34) (all Biolegend, SanDiego, CA) were used for lineage staining. An APC-Cy7- or BV421-labeled streptavidin-antibody (BioLegend) was used for secondary staining together with an APC-anti-cKit (clone 2B8) and a PE-Cy7- or PE-anti-Sca-1 antibody (clone E13-161.7). Cells were analyzed using an BD-Fortessa, LSRII^TM^ or FACSCantoII^TM^ (Becton-Dickinson) cytometer. Analysis was performed using FlowJo^TM^ software (Treestar, Ashland, OR). Cell sorting was performed on a BD FACSAria™ II (Becton-Dickinson).

### Vectors

For RNAi, shRNAs were cloned into a lentiviral pLKO.1_puro vector system for puromycin selection. For HoxA9 overexpression (rescue) experiments, an MSCV-IRES-GFP backbone was used. Lentiviral and retroviral infections were performed as previously described (Schnoeder et al. Blood 2022). Detailed information on vectors and sequences are provided in Supplementary Tables 1 and 2.

### Genome editing by CRISPR/Cas9

**Fig. 1:**
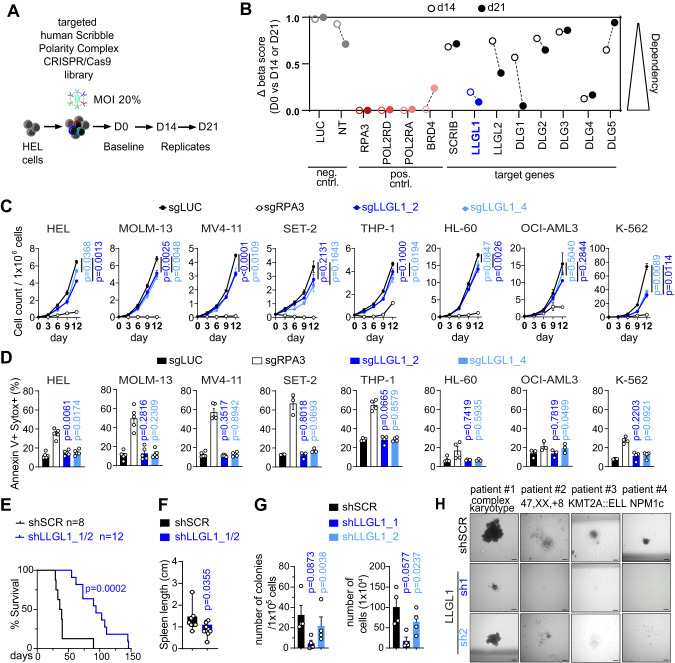
Targeted Scribble polarity complex screen identifies *LLGL1* as a dependency in human AML. **A** Schematic depicting CRISPR/Cas9 negative selection screen strategy. **B** Dependency scores for days 14 and 21 compared to baseline. **C**, **D** Genetic inactivation of *LLGL1* by CRISPR/Cas9-induced knockout of *LLGL1* compared to non-targeting control (sgLUC) in human AML cell lines expressing Cas9. **C** Proliferation assayed by cell counting after trypan blue exclusion. *n* = 3–5 independent experiments; mean ± SD; paired Student’s *t* test. **D** Apoptosis assays using SytoxBlue and Annexin V staining. *n* = 3–5 independent experiments; mean ± SD; paired t-test. **E**, **F** Xenografting of human AML cells in NSGS mice. **E** Survival curves of NSGS recipient mice following transplantation of 1 × 10^5^ MOLM-13 cells following inactivation of *LLGL1* (shLLGL1_1/2; *n* = 12) by RNAi compared with non-targeting control (shSCR; *n* = 8); Mantel–Cox test. **F** Spleen size of recipient animals; mean ± SEM; unpaired t-test. **G**, **H** Colony formation (CFU-) assays of primary human AML following inactivation of *LLGL1* (shLLGL1_1/2) by RNAi compared with non-targeting control (shSCR) in methylcellulose; *n* = 4 independent replicates, paired t-test. **G** Colony count, total cell numbers and **H** Representative pictures of colony morphology. Cytogenetic and molecular genetic aberrations are indicated above. Scale bars = 200 µm; magnification 4×.

Genetic editing by CRISPR/Cas9 was performed as previously described [10, 16] unless otherwise stated. Guide RNAs were designed using the Broad GPP tool [17]. For cloning of sgRNA sequences, the improved-scaffold-pU6-sgRNA-EF1Alpha-PURO-T2A-RFP (ipUSEPR) vector system [18], with puromycin resistance and RFP selection marker was used. Genetic inactivation by CRISPR/Cas9 was performed as published before [15]. HEL cells were transduced with the screen library and selected for 2 days with puromycin following collection of an aliquot as the input reference. Cells were cultured in vitro and samples for sequencing were collected 2 and 3 weeks later (Fig. 1A). The average relative abundance of each sgRNA in the output compared to the input samples was determined. We calculated a depletion score for each sgRNA. The median of 3-4 sgRNAs per gene was used to represent the score of the corresponding gene. Knockdown efficiency was assessed by quantitative real-time PCR (RT-qPCR) 5-7 days post-infection as published before [10]. qPCR primer sequences are listed in the Supplementary Table 3. sgRNA sequences are provided in the Supplementary Tables 4 and 5.

### CRISPR/Cas9 in vitro screen

HEL cells were transduced with the Scribble complex member library at a multiplicity of infection (MOI) of 20%, selected for 2 days with puromycin and an input reference (baseline) was taken at day 4 post-infection. The cells were cultured in vitro for 21 days and replicates 1–4 were taken at day 14 and day 21, respectively. Genomic DNA was isolated using the QIAmp DNA Blood Mini Kit (Qiagen, Hilden Germany), and amplification was performed using specific Illumina primer compatible sequences (Supplementary Table 6). Sequencing was performed at Genewiz (HiSseq, 150 bp, paired end) (Illumina, South Plainfield, NJ, USA). A depletion score for day 14 and day 21 were analyzed compared to the input reference using the MAGeCKFlute [19].

### RNA-sequencing

RNA was isolated from cultured cells using the Qiagen RNeasy Mini kit or TRIZOL as previously described [15, 20]. Subsequently, mRNAs were purified using the “NEBNext® Poly(A) mRNA Magnetic Isolation Module” followed by RNAseq library preparation using the “NEBNext® Ultra™ RNA Library Prep Kit for Illumina®” according to the manufacturer’s instruction. Sequencing was performed on an Illumina NextSeq500 or an Illumina HiSeq2000 (75 bp, single end) (Illumina, South Plainfield, NJ, USA).

### SC-flow cytometry

For the single-cell flow cytometry analysis, bone marrow cells were resuspended in PBS/2%FBS. The staining was performed for 20 minutes at 4 °C with the antibodies mentioned in Supplementary Table 7. After washing, samples were acquired on an Aurora spectral flow cytometer (Cytek Bioscinces, Fremont, CA) equipped with 5 lasers (355 nm, 405 nm, 561 nm, 640 nm) using SpectroFlo version 3.1.0 (Cytek Bioscinces, Fremont, CA). All flow cytometry data were analyzed and exported as compensated channel values with FlowJo^TM^ software (Treestar, Ashland, OR; version 10.8.1).

### Dimensionality reduction of flow cytometry data

Each sample was subset down to 30,000 cells LogNormalization (Seurat function; v4.3.0) was applied before downstream analysis [21]. The compensated channel value for each cell were normalized by total channel values for that cell, multiplied by 10,000 (TP10K), and then log-transformed by log10 (TP10k + 1). After scaling, the dimensionality of the flow data was set to 33 principal components that were used as input for UMAP representation.

### Clustering of flow cytometry data

Following the selection of variable genes and the performance of Principal Component Analysis (PCA), Uniform Manifold Approximation and Projection (UMAP) was applied to visualize the cell population. Subsequently, the cells were clustered using the Louvain algorithm [22] based on a Shared Nearest Neighbor (SNN) graph with a resolution parameter set to 0.29. However, one particular cell cluster was excluded from further analysis as it displayed no detectable expression of any marker genes. Ultimately, these identified clusters were annotated according to their respective cluster markers. The plots were generated using the packages ggplot2 (v3.4.1) and virdis (v0.6.3) in R 4.2.3.

### Statistical analysis

Kaplan–Meier curves were plotted using GraphPad Prism version 9.0 (GraphPad Software, San Diego, CA) using the log-rank test (Mantel–Cox test). Statistical analyses were performed using ANOVA with FDR *p*-value correction for comparing more than two groups or t-test for comparing two groups, unless stated otherwise. Significance of *p*-values in figures are indicated using the following ranges: *p < 0.05; **p < 0.01; ***p < 0.001; ****p < 0.0001. Each dot represents an individual biological replicate.

## Results

### Inactivation of *LLGL1* results in impaired proliferative capacity of human AML

We sought to investigate the context specific effects and functional dependencies of all human Scribble complex homologs in human AML. We performed an arrayed CRISPR/Cas9-based negative selection screen (Fig. 1A and Supplementary Fig. 1A) using the human AML cell line HEL. All positive controls resulted in dropout of the respective clones on days 14 and 21, while negative controls left the infected HEL cells unaffected. Out of the Scribble complex members, depletion of *LLGL1* and *DLG4* (days 14 and 21) and *DLG1* (only day 21) could be detected (Fig. 1B). These findings were rather unexpected as deletion of *LLGL1* had resulted in expansion of normal HSCs and its expression had been correlated with decreased survival in AML [4]. To confirm the CRISPR/Cas9 screening results, we inactivated *LLGL1* by CRISPR/Cas9 induced knockout in 8 different human AML cell lines. All cell lines showed decreased proliferative capacity upon depletion of *LLGL1* (Fig. 1C). This loss of proliferative capacity could not be attributed to induction of apoptosis (Fig. 1D) and was variable between cell lines harboring different driver mutations (Fig. 1C, D, Supplementary Fig. 1B, C). To validate the functional impact of *LLGL1* deletion in human AML cells in vivo, we performed RNAi-mediated deletion of *LLGL1* in MOLM-13 cells and assessed for disease dynamics after transplantation into humanized NSGS mice (Fig. 1E, F, Supplementary Fig. 1D). Inactivation of *LLGL1* reduced disease activity as indicated by reduced spleen size of recipient mice (*p* = 0.0355, Fig. 1F). Moreover, deletion of *LLGL1* delayed disease progression in vivo. When injecting 1 × 10^5^ transduced cells, overall survival was significantly improved (median survival of shSCR: 36 days; shLLGL1: 90 days; *p* = 0.0002) (Fig. 1E). Consistently, inactivation of *LLGL1* in leukemic cells derived from different AML patient-derived xenograft models (PDX) resulted in reduced colony forming capacity in methylcellulose (Fig. 1G, H, Supplementary Fig. 1E). These findings indicate the requirement for *LLGL1* to maintain proliferative capacity in human AML across various genetic subtypes.

### *Llgl1* is required for disease maintenance in AML driven by different oncogenes

**Fig. 2:**
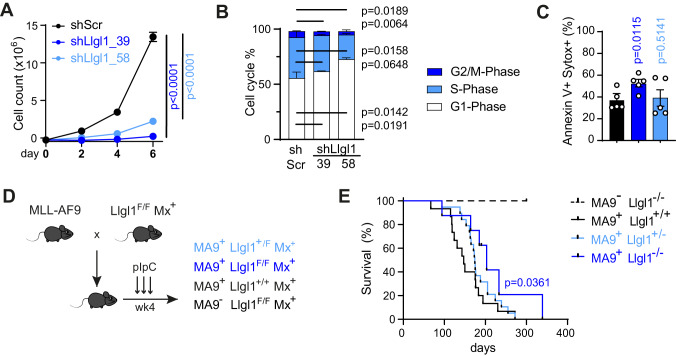
Genetic inactivation of *Llgl1* impairs proliferative capacity of MLL-AF9 driven murine AML. **A**–**C** RNAi-mediated depletion of *Llgl1* with shRNAs targeting *Llgl1* (shLlgl1_39/_58) or non-targeting control (shScr) in murine MLL-AF9 (MA9)-GFP cells. **A** Proliferation assayed by cell counting after trypan blue exclusion. 5 independent experiments, paired t-test. **B** Cell cycle analysis using EdU- and FxCycleViolet-staining in flow cytometry. *n* = 3 independent experiments; ±SD; paired t-test. **C** Apoptosis assay using SytoxBlue and Annexin V staining. *n* = 5 independent experiments; mean ± SEM; paired t-test. **D** Schematic representation of the experimental setup to study the effects of *Llgl1* inactivation using a conventional MLL-AF9 driven knock-in model [12]. pIpC injections for conditional deletion of *Llgl1* were administered intraperitoneally as indicated by arrows. **E**. Survival curves of MA9^−^; Llgl1^−/−^ (*n* = 4), MA9^+^; Llgl1^+/+^ (*n* = 15), MA9^+^; Llgl1^−/−^ (*n* = 8) and MA9^+^; Llgl1^+/−^ (*n* = 19) animals. Mantel–Cox test.

Murine models of AML allow for a more detailed and controlled assessment of functional consequences after inactivation of cell fate determinants such as *Llgl1* as they lack the inter-individual heterogeneity of human samples. Inactivation of *Llgl1* by RNAi with two different shRNAs in murine leukemia induced by retroviral infection with MLL-AF9 resulted in significant decrease of proliferative capacity compared to non-targeting control (*p* < 0.0001; Fig. 2A, Supplementary Fig. 2A). This effect could be attributed to reduced cell cycle activity (Fig. 2B, Supplementary Fig. 2B) rather than induction of apoptosis (Fig. 2C, Supplementary Fig. 2C).

As genetic deletion of *Llgl1* may result in disturbed cell adhesion and division, we sought to validate its function in a model system that allows assessment of functional consequences following conditional *Llgl1* deletion in adult hematopoietic cells [23] and also in established leukemia. In this model, exon 2 is flanked by loxP-sites and genetic deletion of this region results in loss of a functional protein [4, 13]. These conditional *Llgl1* knockout animals were intercrossed with Mx1-Cre^+^ animals and a conventional (straight) MLL-AF9 knock-in model that develops myeloid leukemia with a median latency of 5-6 months [12]. We compared MLL-AF9 negative animals with *Llgl1* inactivation (MA9^−^; Llgl1^F/F^; Mx^+^) to littermate MLL-AF9 positive controls. MLL-AF9 positive animals had either unexcised/wildtype *Llgl1* (MA9^+^; Llgl1^F/F^; Cre^−^ or MA9^+^; Llgl1^+/+^; Mx^+^), a heterozygous state (MA9^+^; Llgl1^+/F^; Mx^+^) or homozygosity of the conditional allele (MA9^+^; Llgl1^F/F^; Mx^+^) (Fig. 2D). Genetic inactivation of *Llgl1* was induced by administration of pIpC at 4 weeks of age. We monitored peripheral blood (PB) counts and distribution of immune cell subsets as well as clinical signs of disease development over time. MLL-AF9 negative animals with conditionally inactivated *Llgl1* did not develop any signs of disease. In contrast, deletion of *Llgl1* in MLL-AF9 positive mice resulted in delayed disease development of heterozygous (median survival: 174 days; *p* = 0.0943) and homozygous (median survival: 203 days, *p* = 0.0361) animals as compared to *Llgl1* wildtype littermate controls (median survival: 147 days) (Fig. 2E). While disease development appeared prolonged in a gene dose dependent manner, we found neither changes in disease penetrance nor significant differences in blood counts or spleen size at timepoints between 64 and 300 days after pIpC injection, when individual animals were sacrificed due to clinically apparent signs of disease.

**Fig. 3:**
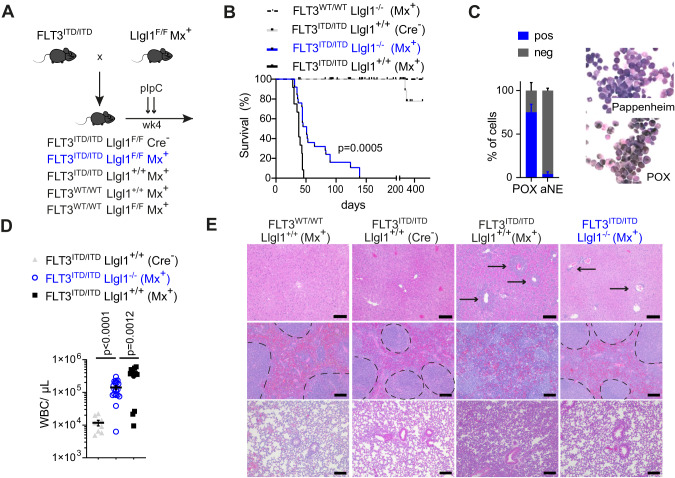
Conditional deletion of *Llgl1* delays development of murine FLT3^ITD/ITD^ AML. **A** Experimental setup to study the effects of *Llgl1* inactivation in FLT3^ITD/ITD^ driven leukemia. pIpC injections for conditional deletion of *Llgl1* were administered intraperitoneally as indicated by arrows. **B** Survival curves of FLT3^WT/WT^; Llgl1^−/−^; Mx^+^ (*n* = 6), FLT3^ITD/ITD^; Llgl1^+/+^; Cre^−^ (*n* = 73), FLT3^ITD/ITD^; Llgl1^−/−^; Mx^+^ (*n* = 25) and FLT3^ITD/ITD^; Llgl1^+/+^; Mx^+^ (*n* = 12), respectively. Mantel–Cox test. **C** Bone marrow cytospins using May–Grünwald/Giemsa or POX staining; scale bars = 10 µm; magnification 40×. **D** White blood count (WBC). Mean ± SEM; unpaired t-test. **E** Histology of liver, spleen and lung. Representative images; arrows indicate leukemic infiltration; spleen white pulp highlighted by dotted line. Scale bars = 100 µm; magnification 10×.

In order to confirm our findings in a second oncogenic model of acute myeloid leukemia, we crossed FLT3-ITD knock-in mice [11] with transgenic animals expressing the interferon inducible Mx1-Cre-recombinase and the conditional *Llgl1* knockout (Fig. 3A). FLT3-ITD is one of the most common recurrent genetic mutations found in patients with AML [24] and FLT3-ITD knockin mice have been a valuable research model for studying the effect of cooperative gene mutations [25]. Most recently, we have shown that - in this model - Cre-expression aggravates the AML disease phenotype [26]. Following induction of Mx1-Cre recombinase by repeated pIpC injections at 4 weeks of age, FLT3^ITD/ITD^; Llgl1^+/+^ animals developed rapid onset of leukemia with a median survival of 37 days (Fig. 3B). Consistent with our findings in the MLL-AF9 driven model, homozygous deletion of *Llgl1* resulted in significant delay of disease progression (median survival FLT3^ITD/ITD^; Llgl1^−/−^; Mx^+^: 51 days; *p* = 0.0005). Although leukemic cells of FLT3^ITD/ITD^; Llgl1^−/−^; Mx^+^ animals showed blast-like appearance with strong expression of myeloperoxidase (Fig. 3C), peripheral white blood counts at disease onset were significantly lower compared to Llgl1^+/+^ controls (*p* = 0.0012; Fig. 3D). Histopathologic analysis of hematopoietic organs showed decreased leukemia infiltration in FLT3^ITD/ITD^; Llgl1^−/−^ animals and a rescued organ architecture (Fig. 3E).

These findings indicate that *Llgl1* is required for proliferative capacity of murine AML irrespective of the underlying driver mutation and its inactivation leads to prolonged disease development in vivo.

### Inactivation of *Llgl1* results in loss of a stemness-associated phenotype

**Fig. 4:**
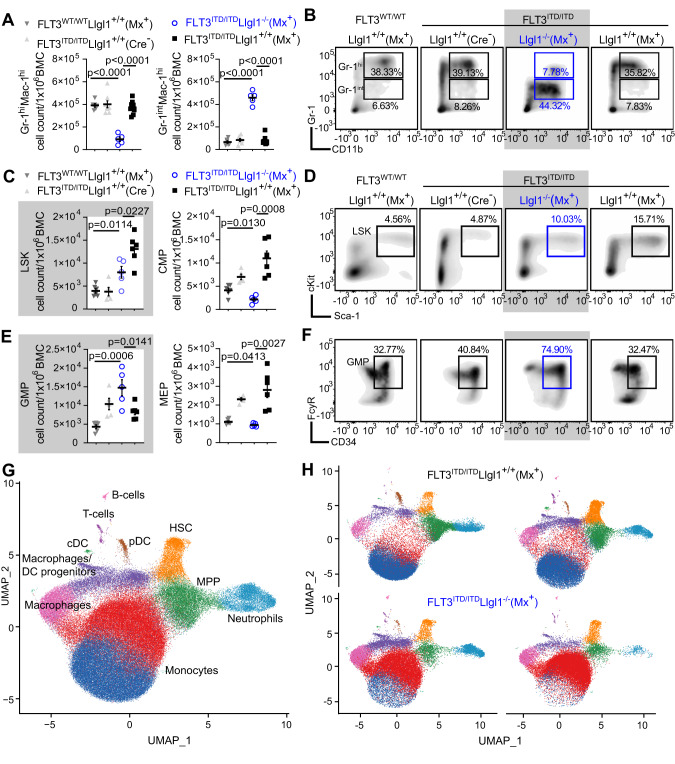
Loss of a stemness-associated immunophenotype of *Llgl1*-deficient AML. **A**–**D** Immuno-phenotyping of FLT3^WT/WT^; Llgl1^+/+^; Mx^+^ (*n* = 6), FLT3^ITD/ITD^; Llgl1^+/+^; Cre^−^ (*n* = 5), FLT3^ITD/ITD^; Llgl1^−/−^; Mx^+^ (*n* = 5) and FLT3^ITD/ITD^; Llgl1^+/+^; Mx^+^ (*n* = 6 mice) bone marrow cells (BMCs). *Llgl1*-deficient genotypes are highlighted in blue. **A** Analysis of mature myeloid cell counts per 1 × 10^6^ BMCs. Mean ± SEM; unpaired t-test. **B** Representative flow cytometric plots of the respective genotypes; mean percentage of parental gate. **C**, **E** Immunophenotypic quantification of immature leukemic cell compartments: LSK (Lin-Sca-1+cKit+), CMP (CD34^+^ FcyR^−^ LK), GMP (CD34^+^ FcyR^+^ LK) and MEP (CD34^−^ FcyR^−^ LK); unpaired t-test. **D**, **F** Representative flow cytometric plots; mean percentage of parental gate. **G** Uniform manifold approximation and projection (UMAP) visualization depicting integrated differentiation trajectories from FLT3^ITD/ITD^; Llgl1^+/+^; Mx^+^ and FLT3^ITD/ITD^; Llgl1^−/−^; Mx^+^ bone marrow cells according to their surface marker expression (34 surface markers, Supplementary Fig. 4; *n* = 2). Clusters are color-coded according to cell type classification (Louvain clustering). **H** Individual UMAP visualization split by FLT3^ITD/ITD^; Llgl1^+/+^; Mx^+^ (upper panel) and FLT3^ITD/ITD^; Llgl1^−/−^; Mx^+^ (lower panel).

When investigating the immunophenotype of FLT3-ITD driven leukemia, variability in myeloid marker expression could be detected. While myeloproliferation induced by FLT3-ITD (FLT3^ITD/ITD^; Cre^−^) and FLT3-ITD-induced acute leukemia (FLT3^ITD/ITD^; Mx^+^) revealed a prominent Gr-1/CD11b high-expressing cell population, deletion of *Llgl1* resulted in abrogation of Gr-1 expression (Gr-1^hi^ expression: 3.7392 ± 0.5731 × 10^5^ BMC in FLT3^ITD/ITD^; Llgl1^+/+^; Mx^+^ versus 0.9182 ± 0.4400 × 10^5^ BMC in FLT3^ITD/ITD^; Llgl1^−/−^; Mx^+^; *p* < 0.0001) (Fig. 4A, B). *Llgl1*-deficient blasts showed reduced expression of Gr-1 (Gr-1^int^: 0.7942 ± 0.3772 × 10^5^ BMC in FLT3^ITD/ITD^; Mx^+^ versus 4.5917 ± 0.5750 × 10^5^ BMC in FLT3^ITD/ITD^; Llgl1^−/−^; Mx^+^; *p* < 0.0001) but remained CD11b positive (Fig. 4A, B). Likewise, alterations in the abundance of immature cell populations could be detected. Previous findings that FLT3-ITD-induced acute leukemia (FLT3^ITD/ITD^; Mx^+^) shows relative expansion of the Lineage- Sca-1+ Kit+ (LSK) compartment [26] compared to FLT3^ITD/ITD^; Cre^−^ animals could be recapitulated (3.968 ± 2.055 × 10^3^ BMC in FLT3^ITD/ITD^; Cre^−^ versus 1.3099 ± 0.3144 × 10^4^ BMC in FLT3^ITD/ITD^; Mx^+^; *p* = 0.0004) (Fig. 4C, D). Here, genetic inactivation of *Llgl1* resulted in a less pronounced expansion of LSK-cells when compared to FLT3^ITD/ITD^; Cre^−^ controls (8.117 ± 2.810 × 10^3^ BMC in FLT3^ITD/ITD^; Llgl1^−/−^; Mx^+^; versus 3.968 ± 2.055 × 10^3^ BMC in FLT3^ITD/ITD^; Llgl1^+/+^; Cre^−^; *p* = 0.0285). In contrast, loss of *Llgl1* expression led to significant expansion of leukemic cells characterized by a more differentiated, granulocyte-monocyte-progenitor (GMP) -like immunophenotype (Lineage−, Kit+, FcgR+, CD34high) (Fig. 4E, F) compared to FLT3^ITD/ITD^; Mx^+^ and FLT3^ITD/ITD^; Cre^−^ animals. Relative abundance of blasts with a GMP-like immunophenotype was more than 2-fold in FLT3^ITD/ITD^; Llgl1^−/−^; Mx^+^ versus FLT3^ITD/ITD^; Llgl1^+/+^; Mx^+^ animals (1.4755 ± 0.4783 × 10^4^ BMC versus 8.306 ± 1.963 × 10^3^ BMC, respectively). Conversely, leukemic cells with a common myeloid progenitor (CMP) or megakaryocyte-erythroid-progenitor (MEP) phenotype appeared reduced in *Llgl1*-deficient leukemic animals (Fig. 4C, E). In order to define the loss of stemness in more detail, we performed multi-parametric single-cell immunophenotyping of bone marrow cells derived from the FLT3-ITD driven leukemia model (Fig. 4G, H, Supplementary Fig. 3). Here, relevant decrease of HSC-like (yellow) and MPP-like (green) blast populations and a shift towards a more monocyte-like population (red). Moreover, the monocytic (and characteristic) phenotype observed in FLT3-ITD driven blasts [11] changes upon inactivation of *Llgl1* (blue to red), indicating a switch in maturation stage. These results indicate immunophenotypic changes of the leukemic cell population and specifically loss of the stemness-associated marker profile.

### Loss of *Llgl1* expression can be rescued by re-expression of *Hox*-genes

**Fig. 5:**
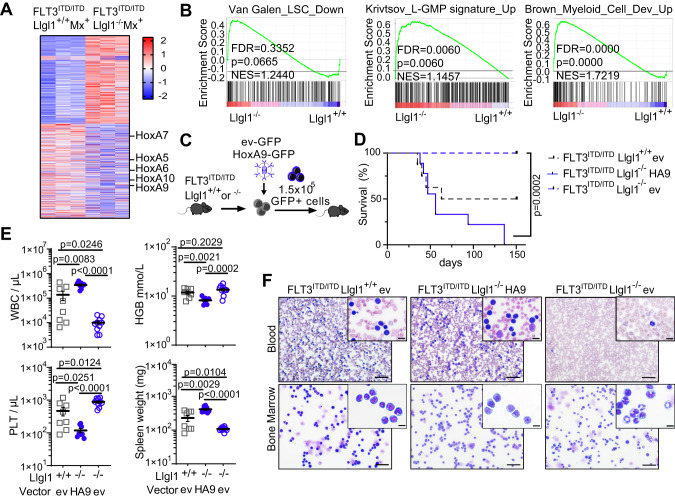
Re-expression of Hoxa9 rescues the disease phenotype of *Llgl1*-deficient FLT3^ITD/ITD^ AML. **A** Heatmap of differentially expressed genes of FLT3^ITD/ITD^; Llgl1^−/−^; Mx^+^ vs FLT3^ITD/ITD^; Llgl1^+/+^; Mx^+^ LSK cells (*n* = 3). Red zones represent higher gene expression (upregulation) and blue zones represent lower gene expression (downregulation). **B** GSEA indicating loss of LSC gene signatures (left panel), enrichment for L-GMP signatures (center) and myeloid cell development (right panel) in *Llgl1*-deficient FLT3^ITD/ITD^ LSKs. NES, normalized enrichment score; FDR, false discovery rate. **C**–**F** Retroviral expression of Hoxa9 in FLT3^ITD/ITD^ leukemia for functional rescue of *Llgl1*-deficient leukemic cells. **C** Schematic of experimental setup. **D** Survival curves of recipient animals of FLT3^ITD/ITD^; Llgl1^+/+^ empty vector (ev) *n* = 8; FLT3^ITD/ITD^; Llgl1^−/−^ Hoxa9 (HA9) *n* = 9; FLT3^ITD/ITD^; Llgl1^−/−^ ev *n* = 10. Shown are 2 independent cohorts; Mantel–Cox-test. **E** White blood count (WBC), hemoglobin (HGB), platelets (PLT) and spleen weight (in mg) of recipient animals; unpaired t-test. **F** Representative smears of peripheral blood and bone marrow cytospins using May–Grünwald/Giemsa; bigger pictures: scale bars = 50 µm; magnification 20×; smaller picture: scale bars = 10 µm; magnification 40×.

In order to assess for transcriptomic changes as a consequence of *Llgl1* deletion, we analyzed sorted Lin-Sca1+Kit+ (LSK) cells from both, FLT3^ITD/ITD^; Llgl1^−/−^; Mx^+^ versus FLT3^ITD/ITD^; Llgl1^+/+^; Mx^+^ animals. *Llgl1* deficient LSK cells showed significant decrease in *HoxA* gene expression, specifically *HoxA5-7* and *HoxA9-10* (Fig. 5A). Gene-set enrichment analysis (GSEA) revealed loss of leukemia stem cell (LSC) associated gene signatures as well as enrichment of L-GMP and myeloid differentiation associated gene sets (Fig. 5B). As loss of *HoxA* gene expression and enrichment of differentiation associated signatures are consistent with the observed loss of a stemness-associated immunophenotype, we sought to investigate whether re-expression of *Hox*-genes could rescue the loss-of-function phenotype. FLT3^ITD/ITD^; Llgl1^−/−^; Mx^+^ or FLT3^ITD/ITD^; Llgl1^+/+^; Mx^+^ LSK cells were retrovirally transduced with a HoxA9-GFP expressing construct (HA9) or empty vector control (ev). 1.5 × 10^5^ GFP+ cells were transplanted at limiting dilution into sublethally irradiated recipient mice (Fig. 5C). As expected, injection of equal numbers (1.5 × 10^5^ GFP+) of empty-vector transduced FLT3^ITD/ITD^; Llgl1^+/+^; Mx^+^ cells induced leukemia in 50% of recipient animals (median survival: 63 days; Fig. 5D). In contrast, injection of *Llgl1*-deficient FLT3^ITD/ITD^; Mx^+^ cells did not result in development of AML in vivo, confirming the loss-of function phenotype. Re-expression of *HoxA9* in *Llgl1*-deficient leukemic cells resulted in a complete phenotypic rescue and resulted in lethal leukemia with 100% penetrance (median survival 56 days, Fig. 5D). Consistently, *HoxA9* re-expression led to significant increase in white blood cell count (*p* < 0.0001), and spleen weight (*p* < 0.0001) as well as significant decrease in platelet numbers (*p* < 0.0001) and hemoglobin concentration (*p* = 0.0002) compared to empty vector control (Fig. 5E). These functional endpoints could also be confirmed by phenotypic analyses of bone marrow and peripheral blood cell morphology. While *Llgl1*-deficient FLT3^ITD/ITD^; Mx^+^ leukemic cells with overexpression of empty vector showed signs of cellular differentiation (Fig. 5F, right panel), expression of *HoxA9* resulted in re-induction of immature cell morphology (Fig. 5F, center), comparable to *Llgl1*-wildtype FLT3^ITD/ITD^; Mx^+^ controls (Fig. 5F, left panel).

Taken together, the phenotype induced by inactivation of *Llgl1* is characterized by a decrease of stemness-associated gene expression signature, including loss of *HoxA* gene expression. Conversely, re-expression of *HoxA9* can rescue this phenotype and results in re-establishment of an immature and aggressive leukemia.

## Discussion

Previously, genetic inactivation of *Llgl1* had been associated with a significant increase in long-term (LT-) HSC numbers and these cells show a competitive advantage when transplanted serially into secondary recipient mice [4]. Moreover, loss of *Llgl1* expression had been associated with inferior survival in datasets of AML and the *Llgl1* knockout gene signature in HSCs correlated with AML gene sets predicting dismal outcome. On the other hand, the tumor suppressor function of *Llgl1* was not conserved in murine models of lymphoid (B- and T-cell) leukemia [9] suggesting that *Llgl1* may act in a highly cell context specific manner. Here, we report on an unbiased targeted CRISPR/Cas9 screening approach to investigate the functional impact of Scribble polarity complex members *SCRIB*, *LLGL1*&2 and *DLG1-5* on cellular function of AML. Loss of *LLGL1* led to depletion of transduced cells. Impairment of proliferative capacity could be confirmed in human AML in vitro and in vivo as well as in two different mouse models of AML. These findings provide first evidence that tumor-suppressor function of *Llgl1* as indicated by the gain of function phenotype in *Llgl1*-deficient HSCs is not conserved in models of acute myeloid leukemia. Conversely, inactivation of *Llgl1* reveals inhibitory effects on disease development and propagation. Likewise, loss of *Llgl1* had not altered the course of murine lymphoid neoplasms induced by constitutive *Notch*, *c-Myc* or *Jak2* expression [9]. These results suggest that the role of *Llgl1* in hematopoietic cells may depend on lineage specificity, type of underlying oncogenic mutations and specific cellular contexts.

Interestingly, inactivation of *Llgl1* resulted in loss of stemness-associated gene expression consistent with acquisition of a more differentiated GMP-like immunophenotype. GMP-like leukemia stem cells have revealed a less aggressive phenotype in MLL-fusion induced models when compared to HSC-derived leukemias [27]. These findings are consistent with the observed delay in *Llgl1*-deficient leukemia development. Although FLT3-ITD driven AML is not among genetic subtypes with the highest levels of *HOX*-gene expression (such as *NPM1*-mutated AML) we found reduced expression of the *HoxA*-gene cluster along with other stemness associated genes. Loss of a stemness-associated gene expression program by deletion of *Llgl1* may indicate its involvement in regulation of cell polarity, which has been previously described in other model systems [28]. Here, disruption of polarity may alter asymmetric cell division (ACD), shift ACD of leukemia stem cells towards symmetric cell division and result in loss of self-renewal capacity. Conversely, gain of a self-renewal associated gene expression signature in normal HSCs [4] may rather be attributed to increased symmetric renewal – in a context specific manner. Impressive work on normal HSCs has recently shown that HSC fitness response to stress depends on signaling molecules Yap1 and Taz, and that deletion of *Yap1* and *Taz* induces loss of HSC quiescence and symmetric self-renewal ability [3]. Moreover, this work provided evidence that Scrib-complex member Scribble and Yap1 coordinate to control Cdc42 activity and HSC fate determination. While the mechanistic role of *Llgl1* deletion in cell fate decisions of LSCs and its potential interaction with Yap-Taz-signaling is clearly beyond the scope of this manuscript, it is tempting to speculate on a cell-context specific role of Llgl1 in maintenance of an immature cell state through interaction with the described pathways.

Taken together, cell-type specific functions of Scribble complex member *Llgl1* could be confirmed in acute leukemia, which may suggest a possible disease specific modulation of cell polarity complexes.

### Supplementary information

Supplementary Figure 1

Supplementary Figure 2

Supplementary Figure 3

Supplementary Figure Legends

Supplementary Tables

## Data Availability

RNA-sequencing data are available through NCBI Gene Expression Omnibus under accession numbers GSE233381.

## References

[1] Humbert PO, Dow LE, Russell SM (2006). The Scribble and Par complexes in polarity and migration: friends or foes?. Trends Cell Biol.

[2] Humbert PO, Grzeschik NA, Brumby AM, Galea R, Elsum I, Richardson HE (2008). Control of tumourigenesis by the Scribble/Dlg/Lgl polarity module. Oncogene.

[3] Althoff MJ, Nayak RC, Hegde S, Wellendorf AM, Bohan B, Filippi MD (2020). Yap1-Scribble polarization is required for hematopoietic stem cell division and fate. Blood.

[4] Heidel FH, Bullinger L, Arreba-Tutusaus P, Wang Z, Gaebel J, Hirt C (2013). The cell fate determinant Llgl1 influences HSC fitness and prognosis in AML. J Exp Med.

[5] Hawkins ED, Oliaro J, Kallies A, Belz GT, Filby A, Hogan T (2013). Regulation of asymmetric cell division and polarity by Scribble is not required for humoral immunity. Nat Commun.

[6] Hawkins ED, Oliaro J, Ramsbottom KM, Newbold A, Humbert PO, Johnstone RW (2016). Scribble acts as an oncogene in Emu-myc-driven lymphoma. Oncogene.

[7] Mohr J, Dash BP, Schnoeder TM, Wolleschak D, Herzog C, Tubio Santamaria N (2018). The cell fate determinant Scribble is required for maintenance of hematopoietic stem cell function. Leukemia.

[8] Beekman R, Valkhof MG, Sanders MA, van Strien PM, Haanstra JR, Broeders L (2012). Sequential gain of mutations in severe congenital neutropenia progressing to acute myeloid leukemia. Blood.

[9] Hawkins ED, Oliaro J, Ramsbottom KM, Ting SB, Sacirbegovic F, Harvey M (2014). Lethal giant larvae 1 tumour suppressor activity is not conserved in models of mammalian T and B cell leukaemia. PLOS One.

[10] Schnoeder TM, Schwarzer A, Jayavelu AK, Hsu CJ, Kirkpatrick J, Dohner K (2022). PLCG1 is required for AML1-ETO leukemia stem cell self-renewal. Blood.

[11] Lee BH, Tothova Z, Levine RL, Anderson K, Buza-Vidas N, Cullen DE (2007). FLT3 mutations confer enhanced proliferation and survival properties to multipotent progenitors in a murine model of chronic myelomonocytic leukemia. Cancer Cell.

[12] Johnson JJ, Chen W, Hudson W, Yao Q, Taylor M, Rabbitts TH (2003). Prenatal and postnatal myeloid cells demonstrate stepwise progression in the pathogenesis of MLL fusion gene leukemia. Blood.

[13] Klezovitch O, Fernandez TE, Tapscott SJ, Vasioukhin V (2004). Loss of cell polarity causes severe brain dysplasia in Lgl1 knockout mice. Genes Dev.

[14] Velasco-Hernandez T, Säwén P, Bryder D, Cammenga J (2016). Potential Pitfalls of the Mx1-Cre system: implications for experimental modeling of normal and malignant hematopoiesis. Stem Cell Rep.

[15] Jayavelu AK, Schnöder TM, Perner F, Herzog C, Meiler A, Krishnamoorthy G (2020). Splicing factor YBX1 mediates persistence of JAK2-mutated neoplasms. Nature.

[16] Perner F, Schnoeder TM, Xiong Y, Jayavelu AK, Mashamba N, Santamaria NT (2022). YBX1 mediates translation of oncogenic transcripts to control cell competition in AML. Leukemia.

[17] Doench JG, Hartenian E, Graham DB, Tothova Z, Hegde M, Smith I (2014). Rational design of highly active sgRNAs for CRISPR-Cas9-mediated gene inactivation. Nat Biotechnol.

[18] Uckelmann HJ, Kim SM, Antonissen NJ, Krivtsov AV, Hatton C, McGeehan GM (2018). MLL-Menin inhibition reverses pre-leukemic progenitor self-renewal induced by NPM1 mutations and prevents AML development. Blood.

[19] Wang B, Wang M, Zhang W, Xiao T, Chen CH, Wu A (2019). Integrative analysis of pooled CRISPR genetic screens using MAGeCKFlute. Nat Protoc.

[20] Schnoeder TM, Perner F, Heidel FHA (2021). JAK of all trades: how global phosphoproteomics reveal the Achilles heel of MPNs. Mol Cell Oncol.

[21] Stuart T, Butler A, Hoffman P, Hafemeister C, Papalexi E, Mauck WM (2019). Comprehensive Integration of Single-Cell Data. Cell.

[22] Stuart T, Butler A, Hoffman P, Hafemeister C, Papalexi E, Mauck WM (2019). Comprehensive integration of single-cell data. Cell.

[23] Hartleben B, Widmeier E, Wanner N, Schmidts M, Kim ST, Schneider L (2012). Role of the polarity protein scribble for podocyte differentiation and maintenance. PLOS One.

[24] Papaemmanuil E, Gerstung M, Bullinger L, Gaidzik VI, Paschka P, Roberts ND (2016). Genomic classification and prognosis in acute myeloid leukemia. N Engl J Med.

[25] Meyer SE, Qin T, Muench DE, Masuda K, Venkatasubramanian M, Orr E (2016). DNMT3A haploinsufficiency transforms FLT3ITD myeloproliferative disease into a rapid, spontaneous, and fully penetrant acute myeloid leukemia. Cancer Discov.

[26] Straube J, Eifert T, Vu T, Janardhanan Y, Haldar R, von Eyss B (2023). Cre recombinase expression cooperates with homozygous FLT3 internal tandem duplication knockin mouse model to induce acute myeloid leukemia. Leukemia.

[27] Krivtsov AV, Figueroa ME, Sinha AU, Stubbs MC, Feng Z, Valk PJ (2013). Cell of origin determines clinically relevant subtypes of MLL-rearranged AML. Leukemia.

[28] Hawkins ED, Russell SM (2008). Upsides and downsides to polarity and asymmetric cell division in leukemia. Oncogene.

