# Supplementary figures and images for: Cell fate determinant Llgl1 is required for propagation of acute myeloid leukemia
Source: Leukemia. 2023 Aug 16;37(10):2027–35. doi: 10.1038/s41375-023-02005-9 (PMC10539176; doi:10.1038/s41375-023-02005-9)

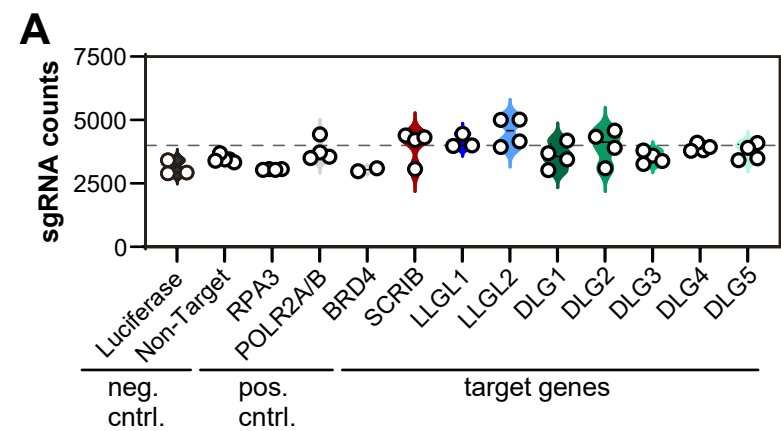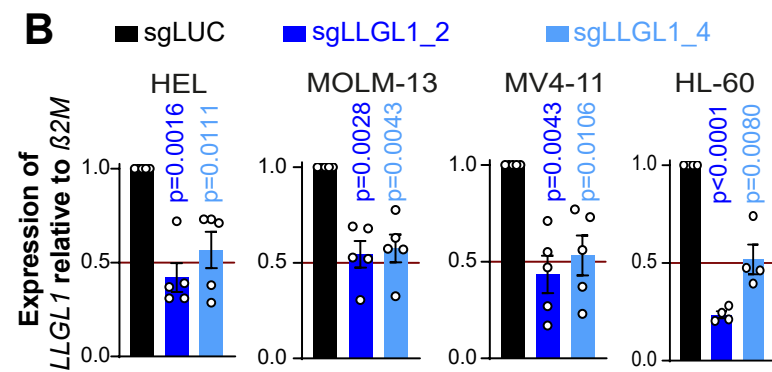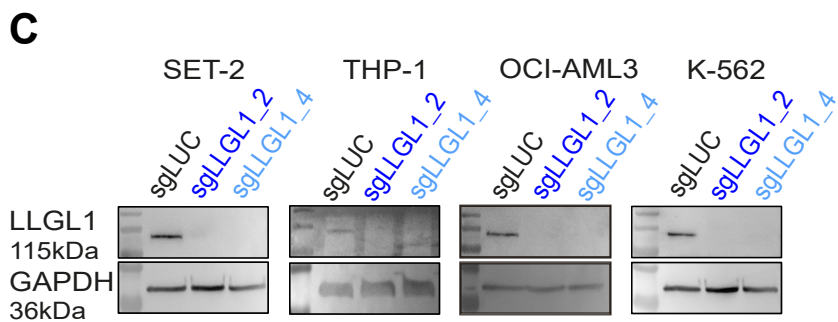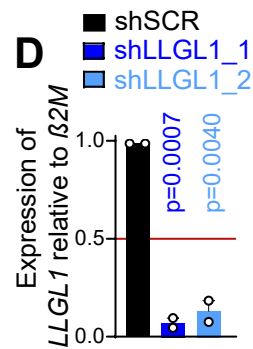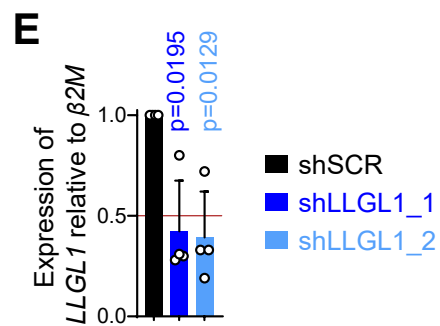

Supplement: Supplementary file 1 — Supplementary Figure 1 [file 41375_2023_2005_MOESM1_ESM.pdf]

**A**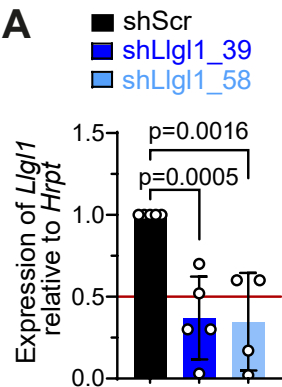**B**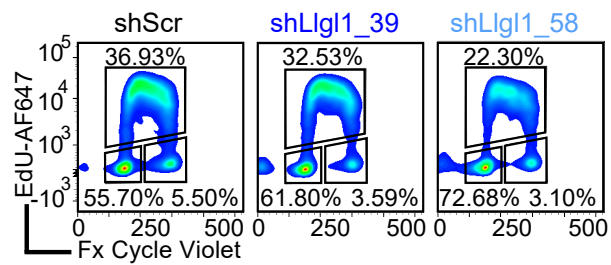**C**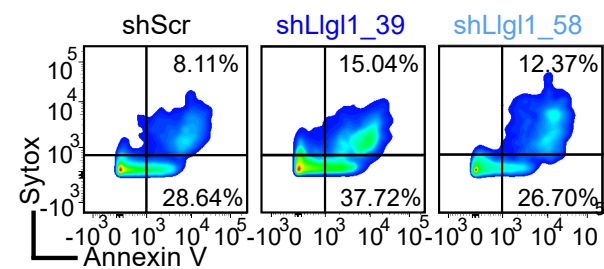

Supplement: Supplementary file 2 — Supplementary Figure 2 [file 41375_2023_2005_MOESM2_ESM.pdf]

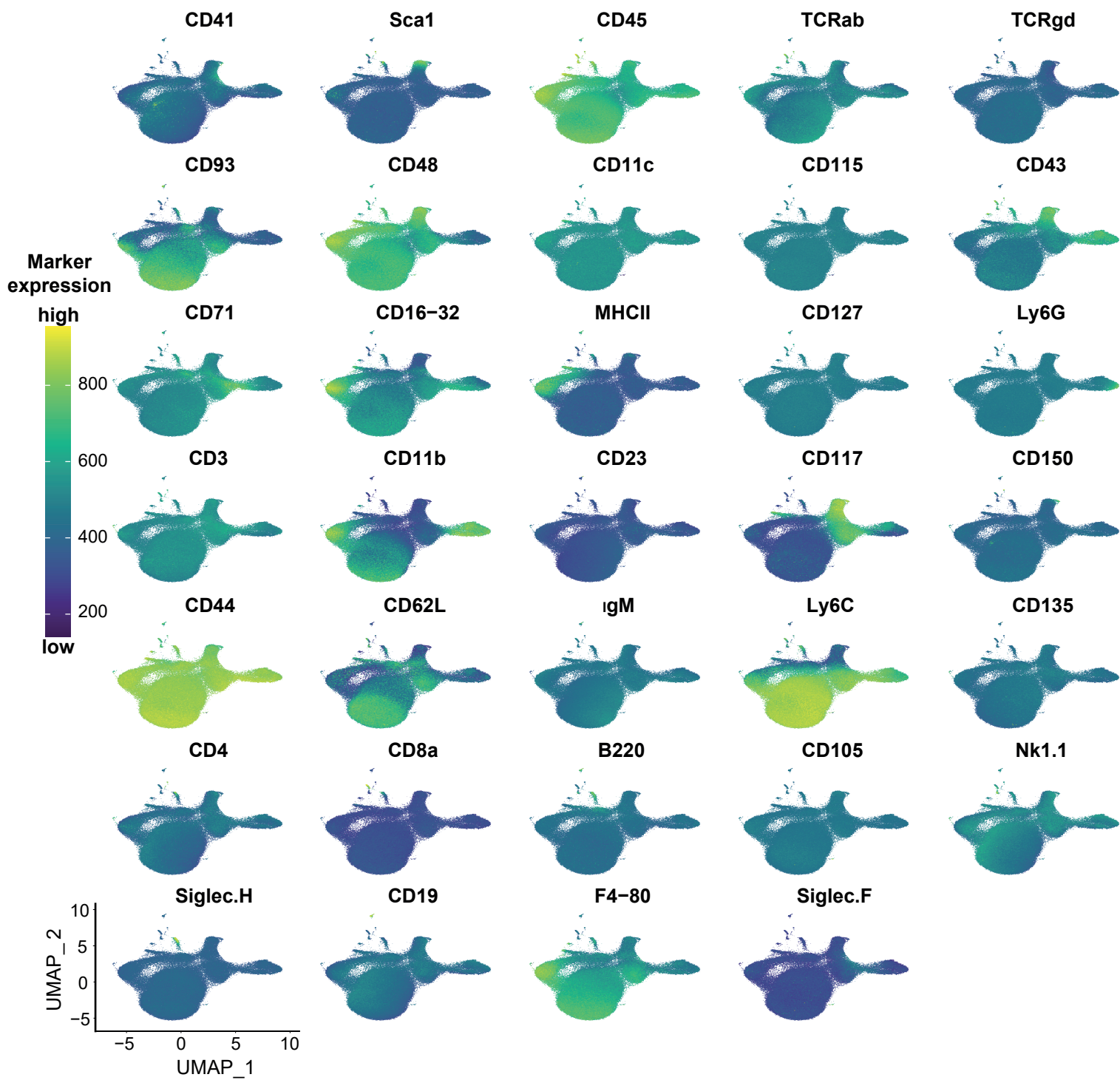

Supplement: Supplementary file 3 — Supplementary Figure 3 [file 41375_2023_2005_MOESM3_ESM.pdf]
