# Supplementary material for: Cell fate determinant Llgl1 is required for propagation of acute myeloid leukemia
Source: Leukemia. 2023 Aug 16;37(10):2027–35. doi: 10.1038/s41375-023-02005-9 (PMC10539176; doi:10.1038/s41375-023-02005-9)
Supplement: Supplementary file 4 — Supplementary Figure Legends [file 41375_2023_2005_MOESM4_ESM.docx]

**Supplementary Figure 1: A** Representation and distribution of the Scribble polarity complex member CRISPR/Cas9 library screen, with negative controls (neg cntrl.: Luciferase, non-targeting), positive controls (pos. cntrl.: *RPA3*, *POLR2A* and *POLR2B*, *BRD4*) and the Scribble polarity complex members (target genes: *SCRIB*, *LLGL1-2*, *DLG1-5*). **B** LLGL1 mRNA expression in different AML cell lines (HEL, MOLM-13, MV-11, HL-60) following CRISPR/Cas9-induced knockout using specific sgRNAs (sgLLGL1_2 or sgLLGL1_4) or a non-targeting control (sgLUC). Data show mean ± SD, n=4-5, paired t-test. **C** Western blot analysis in different cell lines (SET-2, THP-1, OCI-AML3, K-562) upon CRISPR/Cas9 knockout using LLGL1-specific sgRNA (sgLLGL1_2 or sgLLGL1_4) or a non-targeting control (sgLUC). Representative images of 3 independent experiments are shown. **D** mRNA expression of LLGL1 in MOLM-13 cells upon RNAi-induced knockdown using specific shRNAs (shLLGL1_1 or shLLGL1_2) or a non-targeting control (shSCR) by RT-qPCR. **E** mRNA expression of LLGL1 in different AML patient samples upon RNAi-induced knockdown using specific shRNAs (shLLGL1_1 or shLLGL1_2) or a non-targeting control (shSCR) by RT-qPCR, n=4 independent AML patients, paired t-test.

**Supplementary Figure 2: A** mRNA expression of Llgl1 in murine Mll-AF9 rearranged (MA9) cells upon RNAi-induced knockdown using specific shRNAs (shLlgl1_39 or shLlgl1_58) or a non-targeting control (shScr) by RT-qPCR. **B-C** Representative flow cytometry plots of cell cycle (B) and apoptosis (C) assays**.** Shown is the plot for one representative experiment; mean percentages are indicated for B, n=3 independent experiments; C, n=5 independent experiments.

**Supplementary Figure 3:** Marker-panel of multi-parametric single-cell analysis to identify cellular differentiation patterns.
