# Supplementary material for: Cell fate determinant Llgl1 is required for propagation of acute myeloid leukemia
Source: Leukemia. 2023 Aug 16;37(10):2027–35. doi: 10.1038/s41375-023-02005-9 (PMC10539176; doi:10.1038/s41375-023-02005-9)
Supplement: Supplementary file 5 — Supplementary Tables [file 41375_2023_2005_MOESM5_ESM.docx]

**Supplementary material:**

**Supplementary Table 1:** shRNA sequences used for functional assays in this study

| shRNA | Species | Ordering number (Sigma-Aldrich) | Sequence (5‘-3‘) |
| --- | --- | --- | --- |
| shSCR | None | SHC002 | CCGGCAACAAGATGAAGAGCACCAA-  CTCGAGTTGGTGCTCTTCATCTTGTT |
| shLLGL1_1 | *Homo sapiens* | TRCN0000117138 | GCCTATACTTTGCCGACACAT |
| shLLGL1_2 | *Homo sapiens* | TRCN0000117139 | CCCATCAGAATTTGAACGCTT |
| shDLG1_1 | *Homo sapiens* | TRCN0000006101 | CGGGTCAATGACTGTATATTA |
| shDLG1_2 | *Homo sapiens* | TRCN0000006102 | CCCACAAGTATGTATATGAAT |
| shDLG4_1 | *Homo sapiens* | TRCN0000235624 | TATGATGTTGTCTACCTAAAG |
| shDLG4_2 | *Homo sapiens* | TRCN0000235627 | ACGAGAGTGGTCAAGGTTAAA |
| shLLGL2_1 | *Homo sapiens* | TRCN0000116432 | CTGGGTCCTTTGGTCAATGTT |
| shLLGL2_2 | *Homo sapiens* | TRCN0000116433 | CCTGTACTTTGCTGACACCTA |
| shLlgl1_39 | *Mus musculus* | TRCN0000088339 | CGTGGAATATGTGAAGGATTT |
| shLlgl1_58 | *Mus musculus* | TRCN0000305158 | GCCTACACCCTTCTGATTAAA |

**Supplementary Table 2:** Vectors used in this study

| Vector | Used for | Source |
| --- | --- | --- |
| psPAX2 | Lentiviral packaging plasmid | Addgene #12260 |
| pMD2.G | Lentiviral packaging plasmid | Addgene #12259 |
| MSCV-GFP-HoxA9 | Retroviral plasmid | kindly provided by Dr. S. Armstrong (Harvard Medical School, Boston, MA, USA) |
| MSCV-GFP-EV | Retroviral plasmid | kindly provided by Dr. S. Armstrong (Harvard Medical School, Boston, MA, USA) |

**Supplementary Table 3:** RT-qPCR primers used for knockout or knockdown validation

| Target gene | Species | Forward primer sequence (5‘-3‘) | Reverse primer sequence (5‘-3‘) |
| --- | --- | --- | --- |
| Llgl1 | *Mus musculus* | CTGCATTCGTCAGCTATCAC | ACTCAAGGCACTGGAGGCA |
| Hprt | *Mus musculus* | GGACAGGACTGAAAGACTTG | CGTTGACTGATCATTACAGTAGC |
| LLGL1 | *Homo sapiens* | GCTGCTTCGATCCCTACAGTGA | CGGCACATCACTAAGCTCCAG |
| B2M | *Homo sapiens* | TGTGTCTGGGTTTCATCCATCCGA | CACACGGCAGGCATACTCATCTTT |

**Supplementary Table 4:** sgRNA sequences used for functional assays in this study

| Target gene | species | Forward primer sequence (5‘-3‘) | Reverse primer sequence (5‘-3‘) |
| --- | --- | --- | --- |
| sgLUC |  | caccGGATTCTAAAACGGATTACC | aaacGGTAATCCGTTTTAGAATCC |
| sgRPA3 | *Homo sapiens* | caccGGTTGGAAGAGTAACCGCCA | aaacTGGCGGTTACTCTTCCAACC |
| sgLLGL1_2 | *Homo sapiens* | caccCCATTAACAAGATTCTGTGG | aaacCCACAGAATCTTGTTAATGG |
| sgLLGL1_4 | *Homo sapiens* | caccGGTGGGCACAGCCATTATGG | aaacCCATAATGGCTGTGCCCACC |

**Supplementary Table 5:** CRISPR/Cas9 Scribble complex member sgRNA sequences

| Gene name | Used as | sgRNA | Direction | Sequence 5’-3’ |
| --- | --- | --- | --- | --- |
| Luciferase | Negative control | 1 | For | caccGGATTCTAAAACGGATTACC |
|  |  |  | Rev | aaacGGTAATCCGTTTTAGAATCC |
|  |  | 2 | For | caccGATTCTAAAACGGATTACCA |
|  |  |  | Rev | aaacTGGTAATCCGTTTTAGAATC |
|  |  | 3 | For | caccAACGCCTTGATTGACAAGGA |
|  |  |  | Rev | aaacTCCTTGTCAATCAAGGCGTT |
|  |  | 4 | For | caccACAACTTTACCGACCGCGCC |
|  |  |  | Rev | aaacGGCGCGGTCGGTAAAGTTGT |
|  |  | 5 | For | caccACGCTGGGCGTTAATCAGAG |
|  |  |  | Rev | aaacCTCTGATTAACGCCCAGCGT |
|  |  | 6 | For | caccAGCTATTCTGATTACACCCG |
|  |  |  | Rev | aaacCGGGTGTAATCAGAATAGCT |
| Non-targeting | Negative control | 1 | For | caccGGAACGAGGCAGTGACAGGG |
|  |  |  | Rev | aaacCCCTGTCACTGCCTCGTTCC |
|  |  | 2 | For | caccTTCACCGTCCACGTGCGCAT |
|  |  |  | Rev | aaacATGCGCACGTGGACGGTGAA |
|  |  | 3 | For | caccTTAGCCCTCGATTGGTTGCG |
|  |  |  | Rev | aaacCGCAACCAATCGAGGGCTAA |
|  |  | 4 | For | caccCCATCACCGATCGTGAGCCT |
|  |  |  | Rev | aaacAGGCTCACGATCGGTGATGG |
|  |  | 5 | For | caccCGCTAGGTTATTTCGTGGCC |
|  |  |  | Rev | aaacGGCCACGAAATAACCTAGCG |
| RPA3 | Positive control | 1 | For | caccGGTTGGAAGAGTAACCGCCA |
|  |  |  | Rev | aaacTGGCGGTTACTCTTCCAACC |
|  |  | 2 | For | caccGATGAATTGAGCTAGCATGC |
|  |  |  | Rev | aaacGATGAATTGAGCTAGCATGC |
| POLR2A | Positive control | 1 | For | caccAAGCGAATGTCTGTGACGGA |
|  |  |  | Rev | aaacTCCGTCACAGACATTCGCTT |
|  |  | 2 | For | caccCAGGGGGTGATTGAGCGGAC |
|  |  |  | Rev | aaacGTCCGCTCAATCACCCCCTG |
| POLR2D | Positive control | 1 | For | caccTGAGAGTGCAGAGGACGAAC |
|  |  |  | Rev | aaacGTTCGTCCTCTGCACTCTCA |
|  |  | 2 | For | caccTGGGCAAAGGTTGGCCAAAC |
|  |  |  | Rev | aaacGTTTGGCCAACCTTTGCCCA |
| BRD4 | Positive control | 1 | For | caccTAAGATCATTAAAACGCCTA |
|  |  |  | Rev | aaacTAGGCGTTTTAATGATCTTA |
|  |  | 2 | For | caccGGGAACAATAAAGAAGCGCT |
|  |  |  | Rev | aaacAGCGCTTCTTTATTGTTCCC |
| SCRIB | Target gene | 1 | For | caccACACAGCCCCCGATCTCGGG |
|  |  |  | Rev | aaacCCCGAGATCGGGGGCTGTGT |
| Gene name | Used as | sgRNA | Direction | Sequence 5’-3’ |
|  |  | 2 | For | caccAGTCTGCCGCAGGATAGTGA |
|  |  |  | Rev | aaacTCACTATCCTGCGGCAGACT |
|  |  | 3 | For | caccCCGACAATACTAAGCCCCAG |
|  |  |  | Rev | aaacCTGGGGCTTAGTATTGTCGG |
|  |  | 4 | For | caccGCCCACGGTGCATTTCGCAG |
|  |  |  | Rev | aaacCTGCGAAATGCACCGTGGGC |
| DLG1 | Target gene | 1 | For | caccCATCCTACCCACCATACCAC |
|  |  |  | Rev | aaacGTGGTATGGTGGGTAGGATG |
|  |  | 2 | For | caccGGAGGTGCAGCACATAAGGA |
|  |  |  | Rev | aaacTCCTTATGTGCTGCACCTCC |
|  |  | 3 | For | caccTTGAGTCATCTCCAATGTGT |
|  |  |  | Rev | aaacACACATTGGAGATGACTCAA |
|  |  | 4 | For | caccGGGGAAATATGCTCTTGAGG |
|  |  |  | Rev | aaacCCTCAAGAGCATATTTCCCC |
| DLG2 | Target gene | 1 | For | caccATTACGAAGATTATACCAGG |
|  |  |  | Rev | aaacCCTGGTATAATCTTCGTAAT |
|  |  | 2 | For | caccTGGACCAGCAGACCTAAGTG |
|  |  |  | Rev | aaacCACTTAGGTCTGCTGGTCCA |
|  |  | 3 | For | caccGTGCTGTAAACAGGTTCCGG |
|  |  |  | Rev | aaacCCGGAACCTGTTTACAGCAC |
|  |  | 4 | For | caccTGGACCAGCAGACCTAAGTG |
|  |  |  | Rev | aaacCACTTAGGTCTGCTGGTCCA |
| DLG3 | Target gene | 1 | For | caccAAAGATGGAAGGTTGCAAGT |
|  |  |  | Rev | aaacACTTGCAACCTTCCATCTTT |
|  |  | 2 | For | caccAGTCCGATCATAATCAAACA |
|  |  |  | Rev | aaacTGTTTGATTATGATCGGACT |
|  |  | 3 | For | caccGGGGATGATTGAGTCTAACA |
|  |  |  | Rev | aaacTGTTAGACTCAATCATCCCC |
|  |  | 4 | For | caccTGTCATATGAGCCAGTGACA |
|  |  |  | Rev | aaacTGTCACTGGCTCATATGACA |
| DLG4 | Target gene | 1 | For | caccTCCACCTCATTATCACGTCG |
|  |  |  | Rev | aaacCGACGTGATAATGAGGTGGA |
|  |  | 2 | For | caccAGGCGAATTGTGATCCACCG |
|  |  |  | Rev | aaacCGGTGGATCACAATTCGCCT |
|  |  | 3 | For | caccATGGGTCGTCACCGATGTGT |
|  |  |  | Rev | aaacACACATCGGTGACGACCCAT |
|  |  | 4 | For | caccATGTAACAAAGATCATCGAA |
|  |  |  | Rev | aaacTTCGATGATCTTTGTTACAT |
| DLG5 | Target gene | 1 | For | caccCATGCAGCACATCCCCAAAG |
|  |  |  | Rev | aaacCTTTGGGGATGTGCTGCATG |
|  |  | 2 | For | caccGGAGCTCAGGCTACGCTCAG |
|  |  |  | Rev | aaacCTGAGCGTAGCCTGAGCTCC |
| DLG5 | Target gene | 3 | For | caccTGTGGGAGACAGGATCGTTG |
|  |  |  | Rev | aaacCAACGATCCTGTCTCCCACA |
|  |  | 4 | For | caccTTTGTCACTAAAGTGGACAA |
|  |  |  | Rev | aaacTTGTCCACTTTAGTGACAAA |
|  |  | 5 | For | caccAGGGAATGGCTTATGCAGAA |
|  |  |  | Rev | aaacTTCTGCATAAGCCATTCCCT |
| LLGL1 | Target gene | 1 | For | caccAGCCAATGAGAATCTTTGTG |
|  |  |  | Rev | aaacCACAAAGATTCTCATTGGCT |
|  |  | 2 | For | caccCCATTAACAAGATTCTGTGG |
|  |  |  | Rev | aaacCCACAGAATCTTGTTAATGG |
|  |  | 3 | For | caccGGAGCTTAGTGATGTGCCGG |
|  |  |  | Rev | aaacCCGGCACATCACTAAGCTCC |
|  |  | 4 | For | caccGGTGGGCACAGCCATTATGG |
|  |  |  | Rev | aaacCCATAATGGCTGTGCCCACC |
| Gene name | Used as | sgRNA | Direction | Sequence 5’-3’ |
| LLGL2 | Target gene | 1 | For | caccCCATCTCGAACACACGCCGG |
|  |  |  | Rev | aaacCCGGCGTGTGTTCGAGATGG |
|  |  | 2 | For | caccCCGGCGGAATGACTGACGCA |
|  |  |  | Rev | aaacTGCGTCAGTCATTCCGCCGG |
|  |  | 3 | For | caccCATGCCACGGGCCAGCTACG |
|  |  |  | Rev | aaacCGTAGCTGGCCCGTGGCATG |
|  |  | 4 | For | caccGGTACTGGAACTGAATGACG |
|  |  |  | Rev | aaacCGTCATTCAGTTCCAGTACC |

**Supplementary Table 6:** P5 (forward) and P7 (reverse) primer used for Illumina sequencing

| Primer name | Full primer sequence 5’-3’ |
| --- | --- |
| P5_for_1 | AATGATACGGCGACCACCGAGATCTACACTCTTTCCCTACACGACGCTCTTCCGATCTTTGTGGAAAGGACGAAACACC |
| P5_for_2 | AATGATACGGCGACCACCGAGATCTACACTCTTTCCCTACACGACGCTCTTCCGATCTCTTGTGGAAAGGACGAAACACC |
| P5_for_3 | AATGATACGGCGACCACCGAGATCTACACTCTTTCCCTACACGACGCTCTTCCGATCTGCTTGTGGAAAGGACGAAACACC |
| P5_for_4 | AATGATACGGCGACCACCGAGATCTACACTCTTTCCCTACACGACGCTCTTCCGATCTAGCTTGTGGAAAGGACGAAACACC |
| P5_for_5 | AATGATACGGCGACCACCGAGATCTACACTCTTTCCCTACACGACGCTCTTCCGATCTCAACTTGTGGAAAGGACGAAACACC |
| P5_for_6 | AATGATACGGCGACCACCGAGATCTACACTCTTTCCCTACACGACGCTCTTCCGATCTTGCACCTTGTGGAAAGGACGAAACACC |
| P7_ rev_1 | CAAGCAGAAGACGGCATACGAGATCGGTTCAAGTGACTGGAGTTCAGACGTGTGCTCTTCCGATCTACACGACATCACTTTCCCAG |
| P7_ rev_2 | CAAGCAGAAGACGGCATACGAGATGCTGGATTGTGACTGGAGTTCAGACGTGTGCTCTTCCGATCTACACGACATCACTTTCCCAG |
| P7_ rev_3 | CAAGCAGAAGACGGCATACGAGATTAACTCGGGTGACTGGAGTTCAGACGTGTGCTCTTCCGATCTACACGACATCACTTTCCCAG |
| P7_ rev_4 | CAAGCAGAAGACGGCATACGAGATTAACAGTTGTGACTGGAGTTCAGACGTGTGCTCTTCCGATCTACACGACATCACTTTCCCAG |
| P7_ rev_5 | CAAGCAGAAGACGGCATACGAGATATACTCAAGTGACTGGAGTTCAGACGTGTGCTCTTCCGATCTACACGACATCACTTTCCCAG |
| P7_ rev_6 | CAAGCAGAAGACGGCATACGAGATGCTGAGAAGTGACTGGAGTTCAGACGTGTGCTCTTCCGATCTACACGACATCACTTTCCCAG |

Green: P5 or P7 flow cell attachment sequence

Purple: Stagger region (P5) or Barcode (P7), respectively

Blue: Illumina sequencing primer binding sequence

Yellow: Vector binding sequence

**Supplementary Table 7**: Flow cytometry antibodies used for single-cell immunophenotyping

| Surface marker | Fluorophore | Ordering No. | Clone | Lot no. | Dilution | Manufacturer |
| --- | --- | --- | --- | --- | --- | --- |
| CD48 | BUV395 | 740236 | HM48-1 | 1112980 | 1:200 | Biolegend |
| CD11c | BUV496 | 750483 | HL3-1 | 1355903 | 1:200 | Biolegend |
| CD115 | BUV563 | 748478 | CSF-1R | 1355893 | 1:200 | Biolegend |
| CD43 | BUV615 | 752307 | S7 | 1355907 | 1:300 | Biolegend |
| CD71 | BUV661 | 741481 | C2 | 1355857 | 1:1000 | Biolegend |
| Surface marker | Fluorophore | Ordering No. | Clone | Lot no. | Dilution | Manufacturer |
| CD16/32 | BUV737 | 612783 | 2.4G2 | 2101862 | 1:500 | Biolegend |
| MHCII | BUV805 | 748844 | M5/114.15.2 | 1355894 | 1:800 | Biolegend |
| CD127 | BV421 | 562959 | SB/199 | 1321701 | 1:50 | Biolegend |
| Siglec F | F-SB436 | 62-1702-82 | 1RNM44N | 2547850 | 1:100 | Thermo Fisher |
| CD105 | PB | 120411 | MJ7/18 | B303063 | 1:200 | Biolegend |
| Ly6G | BV480 | 746448 | A18 | 1355887 | 1:1000 | Biolegend |
| CD3 | BV510 | 100234 | 30-F11 | B361776 | 1:50 | Biolegend |
| CD11b | BV605 | 101237 | M1/70 | B316880 | 1:1000 | Biolegend |
| CD23 | BV650 | 740456 | B3B4 | 1355848 | 1:400 | BD Biosciences |
| CD117 | BV711 | 105835 | 2B8 | B311372 | 1:500 | Biolegend |
| CD150 | BV785 | 115937 | TC15-12F12.2 | B340823 | 1:500 | Biolegend |
| Nk1.1 | PerCP | 108725 | PK136 | B359229 | 1:100 | Biolegend |
| CD93 | BB700 | 742187 | AA4.1 | 2314807 | 1:800 | BD Biosciences |
| SiglecH | PerCPeF710 | 46-0333-82 | eBio440c | 2423776 | 1:1000 | Thermo Fisher |
| CD62L | PE | 161204 | W18021D | B359280 | 1:1000 | Biolegend |
| F4/80 | Sparg YG 593 | 157311 | QA17A29 | B340434 | 1:500 | Biolegend |
| CD135 | PE Dazzle594 | 562537 | A2F10.1 | 2265804 | 1:100 | BD Biosciences |
| CD4 | PE-Fire640 | 100481 | GK1.5 | B343351 | 1:1000 | Biolegend |
| IgM | PE-Cy5 | 406544 | RMM-1 | B313535 | 1:500 | Biolegend |
| CD8a | PE-Fire 700 | 100792 | 53-6.7 | B318676 | 1:1000 | Biolegend |
| Ly6C | PE-Cy7 | 128018 | HK1.4 | B331355 | 1:500 | Biolegend |
| B220 | PE -Fire810 | 103287 | RA3-6B2 | B341831 | 1:500 | Biolegend |
| CD41 | APC | 133914 | MWReg30 | B377160 | 1:200 | Biolegend |
| TCRab | AF647 | 109218 | H57-597 | B335129 | 1:300 | Biolegend |
| CD19 | Spark NIR | 115568 | 6D5 | B344650 | 1:500 | Biolegend |
| TCRgd | R718 | 751919 | GL3 | 1355905 | 1:300 | BD Biosciences |
| (live/dead) | Zombie NIR | 566349 |  |  | 1:100 | Biolegend |
| Surface marker | Fluorophore | Ordering No. | Clone | Lot no. | Dilution | Manufacturer |
| Sca1 | APC-Cy7 | 560654 | D7 | 325565 | 1:1000 | BD Biosciences |
| CD45 | APC-Fire810 | 103174 | 30-F11 | B359177 | 1:500 | BD Biosciences |
| Brilliant stain buffer |  | 566349 |  |  | 1:5 | BD Biosciences |
